# Preparation and Characterization of Biochars from *Eichornia crassipes* for Cadmium Removal in Aqueous Solutions

**DOI:** 10.1371/journal.pone.0148132

**Published:** 2016-02-16

**Authors:** Feng Li, Kaixuan Shen, Xiaolin Long, Jiasheng Wen, Xiaojie Xie, Xiangyun Zeng, Yanyan Liang, Yansha Wei, Zefeng Lin, Wenrou Huang, Ruida Zhong

**Affiliations:** 1 School of Civil Engineering, South China University of Technology, Guangzhou, 510640, China; 2 State Key Laboratory of Subtropical Building Science, South China University of Technology, Guangzhou, 510640, China; São Paulo State University, BRAZIL

## Abstract

The study investigated the preparation and characterization of biochars from water hyacinth at 300°C to 700°C for cadmium (Cd) removal from aqueous solutions. The adsorption process was dominated by oxygen-containing functional groups with irregular surfaces via esterification reactions. Furthermore, the mineral components in the biochars also contributed to Cd absorption through precipitation. Parameters such as the effects of solution pH, contact time, and initial concentration were studied. The optimum pH value was observed at 5.0, in which nearly 90% of Cd was removed. The maximum Cd adsorption capacities based on the Langmuir isotherm were calculated at 49.837, 36.899, and 25.826 mg g^−1^. The adsorption processes of the biochars followed the pseudo-second-order kinetics, with the equilibrium achieved around 5 h. The biochar from *E*. *crassipes* is a promising adsorbent for the treatment of wastewater, which can in turn convert one environmental problem to a new cleaning Technology.

## Introduction

Biochars are carbonaceous residues from the pyrolysis of biomass in an oxygen-limited atmosphere that can be applied in the safe and long-term sequestration of carbon in the environment [1,2]. The application of biochars has attracted increased attention because of the potential of this material in global warming mitigation, soil fertility improvement, pollution remediation, and agricultural waste recycling[3]. Compared with activated carbon, biochars have high adsorption capacities for organic compounds and heavy metals from aqueous solutions[4,5].

However, the high cost of biochars limits their use as adsorbents in wastewater treatment. Thus, deriving biochars from cheap and locally available waste materials is needed to develop a feasible removal technologies[6]. Several studies have explored the production of biochars from feedstock such as sewage sludge, industrial wastes, plant residues, and animal manures [4,7–9]. Despite the benefits of biochar as a sorbent for contaminant management in soil and water, there is still concern about the environmental impacts of potentially excessive levels of heavy metals in biochars since feedstock can be high in heavy metal contents (such as Cu, Cd and Pb)[1]. Therefore the safe use of biochar as soil or water amendments should be ensured by deriving from feedstock less polluted by heavy mental.

To the best of our knowledge, only a few studies have focused on the characteristics of biochars from *Eichornia crassipes* (water hyacinth)[10]. The aggressive invasion of *E*. *crassipes* destroys many ecologically and economically important water bodies and productive wetlands by starving the water of oxygen and destroying native biodiversity[11]. As a result of its high reproductive rate and dispersion, *E*. *crassipes* has been identified by the International Union for Conservation of Nature as one of the 100 most cumbersome invasive species and one of the top 10 worst weeds in the world[12]. Currently, the main treatment method for *E*. *crassipes* is its physical removal from water bodies; however, this treatment has minimal effect[11]. Due to the property of excessive reproduction in unhealthy aquatic ecosystems, *E*. *crassipes* can serve as potential ecological indicator for heavy metal contaminants either at low or high level contamination. Besides, the biomass of *E*. *crassipes* possesses strong adsorption capacity because of its high cellulose content and functional groups, viz. carboxyl (–COO-) and hydroxyl (–OH)[12]. Such huge biomass is speculated to be applicable in wastewater treatment through the conversion of *E*. *crassipes* into biochars. This application turns this invasive weed species into a valuable resource and may represent a substantial method for the management and utilization of this species[10]. Cd is a highly toxic heavy metal. The major sources of Cd contamination are anthropogenic activities, such as the manufacture of alloys, batteries, pigments, and plastics; and mining and smelting processes[13]. The excessive intake of Cd causes anemia, hypertension, muscular cramps, osteoporosis, and renal damage in humans [14]. Hence, wastewater containing high concentrations of Cd must be treated properly before discharge.

The main objective of the present study is to investigate the preparation and characterization of biochars from water hyacinth at 300°C, 500°C and 700°C for Cd removal from aqueous solutions. The mechanisms responsible for the Cd removal capabilities of three biochars were determined using the Brunauer–Emmett–Teller (BET) surface area, Fourier transform infrared spectroscopy (FTIR), scanning electron microscopy (SEM), and X-ray diffraction (XRD). The effects of pH values and solution pH, contact time, and initial concentration on Cd adsorption were also investigated.

## Materials and Methods

### 2.1 Biochar preparation

Fresh *E*. *crassipes* were collected from a stream in Nansha District, Guangzhou, China (lat 22°46′41.24″ N, long 113°35′14.10″ E). The samples were washed with deionized water, dried in an oven at 80°C for 24 h, and grounded through a 0.150 mm sieve. The grounded samples was tightly placed in a porcelain combustion boat, covered with a fitting lid, and then pyrolyzed at various temperatures (i.e., 300°C, 500°C, and 700°C) in a tube furnace (NanDa KTL1400)under oxygen-limited conditions for 2 h. The pyrolysis temperature was programmed at a rate of 10°C/min until the desired values were reached. The biochar samples are hereafter referred to as WH300, WH500, and WH700; the prefixes “WH” are the abbreviated form of *Eichornia crassipes*, and the number following “WH” represents the pyrolysis temperature. All the biochar samples were stored in dark glass bottles before use.

Since the study did not involve animal’s studies, relevant guidelines followed and the committees that approved the study were not required to be identified. The study was carried out on private land, and we have obtained permission from the owner of the land to conduct the study on this site. In addition, the field studies did not involve endangered or protected species. We are responsible for the above statement.

### 2.2 Biochar characterization

Ash content was measured by heating the samples for four hours at 800°C in a tube furnace under air. Biochar yield was determined as the ratio between the biochars and the dry weight of the water hyacinth biomass. Elemental (C, H, N, and O) analyses were conducted in duplicate using an elemental analyzer (MicroCube, Elementar, Germany). The atomic ratios of H/C and (O+N)/C were calculated to evaluate the aromaticity and polarity of the biochars, respectively.

Specific surface areas (S_BET_) were measured through a multipoint BET analysis of the adsorption data points with relative pressures (p/p_0_) between 0.05 and 0.3. The pore properties of the biochars were determined via nitrogen adsorption–desorption at −196°C on a Micromeritics ASAP 2020M surface area analyzer. The total pore volume (V_total_) was estimated from a single N_2_ adsorption point at a N_2_ relative pressure of 0.97. The microporous surface areas (S_micro_) and microporous volumes (V_micro_) of the biochars were obtained with the t-plot method [15]. The average pore sizes (Dap) were calculated from the measured values of S_BET_ and V_total_ [15]. In order to elucidate the pore properties of the resulting biochar, the porous texture was examined by SEM observation. SEM imaging analysis was conducted using a ZEISS-Merlin scanning microscope operated at a 5 kV accelerating potential.

FTIR was employed to identify the functional groups on the biochar surfaces. Prior to the FTIR analyses, all the biochar samples were oven-dried at 70°C for 24 h. To obtain the observable absorption spectra, the samples were prepared by mixing biochar with spectroscopic grade KBr (KBr/ biochar ratio is 100:1) and then compressing the mixture into pellets. The FTIR spectra were obtained between 4,000 and 400 cm^−1^ by using a Bruker Vector 33 spectrometer at a resolution of 4.0 cm^−1^.

The XRD patterns of the biochars were obtained using a Rigaku D/max-3A diffractometer operating with a Cu Kα (λ = 1.541 nm) radioactive source, and the samples were scanned in the range of 3° to 70°. The obtained XRD patterns were analyzed with the Jade 6.0 software to remove the background radiation.

### 2.3 Biochar adsorption experiments

Adsorption experiments were conducted to determine the potential of the biochars to adsorb Cd from aqueous solutions. The period of time between fabrication and use of biochar in adsorption experiments is within three days. All the Cd solutions were prepared before use by diluting the stock solution to the required initial concentration with deionized water. The pH was adjusted to the desired values with 1 M HNO_3_ or 1 M NaOH solution.

The effect of pH on the Cd sorption of the biochars was investigated by varying the solution pH from 1.0 to 6.0 with Cd at 2.0 mg L^−1^ and biochar at 1 g L^-1^. The kinetic experiments were conducted in a series of 25 mL Erlenmeyer flasks by mixing 0.01 g of biochar with 10 mL 0.01 M NaNO_3_ containing 2.0 mg L^−1^ Cd. The mixture pH was adjusted to 5.0. Then, the mixture was agitated on a horizontal shaker (Peiying THZ-C) at 160 rpm in the dark at 25°C. The agitation times were predetermined as 5, 10, and 30 min; and 1, 2, 4, 8, 10, 12, and 24 h. For the adsorption isotherm experiments, 0.01 g of each biochar was added to 10 mL of Cd with desired initial concentrations ranging from 0.01 mg L^−1^ to 50.00 mg L^−1^. The solution pH was adjusted to 5.0, and the ionic strength was adjusted to 0.01 M NaNO_3_[16]. The mixture was shaken at 160 rpm for 24 h at 25°C.

The solutions after Cd adsorption were centrifuged at 4,000 rpm for 15 min. The suspensions were filtered through 0.22 mm filters, and the Cd concentrations of the filtrate were measured with an atomic absorption spectrophotometer (Hitachi Z-2700) equipped with hollow cathode lamps and nebulizer (Hitachi Z-2010). Cd sorption was calculated from the difference between the initial solution concentration and the final solution concentration. All the adsorption experiments were conducted in duplicate, and the results were expressed as the average values of the samples.

To simulate the adsorption isotherms of Cd, the Langmuir (Eq 1) and Freundlich isotherms (Eq 2) were used in fitting the experimental data. The Langmuir model assumes that monolayer adsorption occurs on homogeneous surfaces with no interactions among adsorbed ions. The Langmuir model is often used to describe adsorption capabilities and reflect the absorption equilibrium process. The Freundlich isotherm model assumes that multilayer adsorption occurs on heterogeneous surfaces and that the adsorption amount increases infinitely with increasing concentration. Moreover, the Freundlich isotherm model provides information on chemisorptions on heterogeneous surfaces.

$$\text{Langmuir}:\;q_{e}=q_{\text{max}}\frac{K_{L}C_{e}}{1+K_{L}C_{e}}$$ (1)

$$\text{Freundlich}:\;q_{e}=K_{F}C_{e}^{n}$$ (2)

where q_e_ (mg g^−1^) is the equilibrium Cd adsorption capacity in the solution, q_max_ (mg g^−1^) is the maximum capacity, C_e_ (mg L^−1^) is the equilibrium concentration of Cd, *n* is the Freundlich constant related to adsorption intensity, and K_L_ (L mg^−1^) and K_F_ (mg L^−1^/*n* L^−1^/*n* g^−1^) represent the adsorption constants for the Langmuir and Freundlich models, respectively.

Further kinetic investigation into Cd sorption was performed to describe the adsorption mechanism and assess the efficiency of Cd adsorption. Pseudo and Elovich models (Eq 5) were used to simulate the adsorption kinetics of Cd with respect to the three biochars.

First-order (Eq 3) and second-order (Eq 4)equations describe the kinetics of solid solution systems based on mononuclear and binuclear adsorption, respectively, with respect to sorbent capacity. The pseudo-second-order kinetic model assumes that adsorption capacity is proportional to the number of active sites occupied on the adsorbent. The Elovich kinetic model is an empirical equation that considers the contribution of desorption, which is widely used to describe the sorption of pollutants from aqueous solutions by assuming a high heterogeneity of the adsorbent surface.

$$\text{First-order equation}:\;\frac{\text{d}q_{t}}{dt}=k_{1}(q_{e}-q_{t})$$ (3)

$$\text{Second-order equation}:\;\frac{\text{d}q_{t}}{dt}=k_{2}{\left(q_{e}-q_{t}\right)}^{2}$$ (4)

$$\text{Elovich model}:\;\frac{\text{d}q_{t}}{dt}=\alpha \;\text{exp}(-\beta q_{t})$$ (5)

where q_t_ and q_e_ are the amount of Cd adsorbed at time *t* and at equilibrium, respectively (mg kg^−1^), and k_1_ and k_2_ are the first-order and second-order apparent adsorption rate constants (h^−1^ and kg mg^−1^ h^−1^), respectively. Furthermore, α (mg g^−1^ h^−1^) is the initial Elovich adsorption rate, and β (g mg^−1^) is the Elovich desorption constant related to the extent of the surface coverage and activation energy for chemisorptions.

## Results and Discussion

### 3.1 Characterization of biochars

The physicochemical properties of WH300–WH700 are shown in Table 1. Biochar yield declined as pyrolysis temperature increased. The pH value of the biochars increased from 7.98 to 11.54, which is consistent with the enhanced ash content as the temperature increased from 300°C to 700°C. All the pH measurements were performed in triplicate and the standard deviation (statistics) of pH values were 0.10214, 0.10536, and 0.10817 respectively, which were obtained with SPSS. The carbon content increased from 47.17% to 51.34%, whereas the oxygen, nitrogen, and hydrogen contents decreased from 47.13% to 46.83%, 0.73% to 2.12%, and 1.10% to 3.580%, respectively. The polarity index (O+N)/C ratios decreased with the pyrolysis temperature, thereby indicating a reduction in the surface polar functional groups[17]. As an indication of the degree of aromaticity, the H/C ratio of 0.257 for WH700 suggested that the biochar was highly carbonized with a highly aromatic structure.

**Table 1:** The yields, pH values, chemical compositions and atomic ratios of biochars produced from *E*. *crassipes* at different pyrolytic temperatures.

Biochar	Yield/%	pH	Ash/%	C/%	H/%	N/%	O/%	(O+N)/C	O/C	H/C
**WH300**	46.59	7.98	15.89	47.17	3.58	2.12	47.13	0.788	0.749	0.911
**WH500**	34.97	10.96	27.18	53.39	1.99	1.82	42.80	0.630	0.601	0.447
**WH700**	28.20	11.54	37.25	51.34	1.10	0.73	46.83	0.696	0.684	0.257

**Fig 1:**
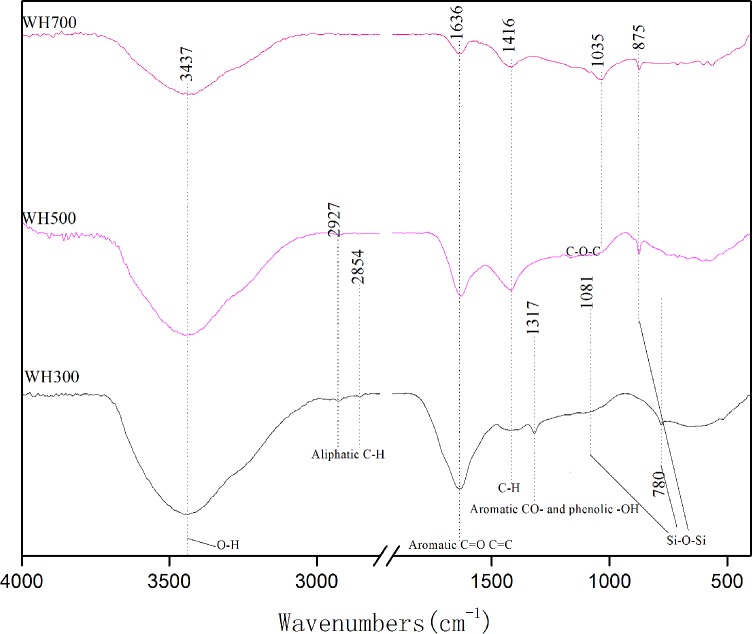
FTIR spectra of biochars pyrolyzed at different temperature.

The FTIR spectra of the biochars (WH300–WH700) are shown in Fig 1. The band at 3,437 cm^−1^ represented the stretching vibrations of the OH groups, which could be attributed to the adsorbed water on the biochar. The asymmetric (2,927 cm^−1^) and symmetric (2,854 cm^−1^) C–H stretching bands were associated with aliphatic functional groups and were observed in WH300 and WH500 but not in WH700, thus indicating a decreased non-polar aliphatic fraction. The bands around 1,636 cm^−1^ were attributed to the aromatic C = O and C = C and diminished as the pyrolysis temperature increased. The C = O functional groups showed very high coordination with the heavy metals. The bands at around 1,317 cm^−1^ were associated with the phenolic -OH or aromatic CO- stretching vibrations. The polar groups (-OH and CO-) significantly decreased when heated to 500°C. In short, the functional groups observed on the biochars included CH, OH, C = O, and CO, all of which may have contributed to the Cd sorption. On the basis of these results, we can deduce that the good sorption properties of the adsorbent toward Cd can be attributed to the presence of the functional groups in the adsorbent.

As shown in Table 2, the surface areas and pore volumes increased significantly as the pyrolysis temperature increased. The surface areas (S_BET_) of WH300 were extremely low (3.5150 m^2^ g^−1^), and the S_BET_ of WH500 increased slightly at 6.7131 m^2^ g^−1^ because of the removal of OH, aliphatic CO, and ester CO groups from the outer surfaces of the biochar. The S_BET_ of WH700 increased sharply to 175.4568 m^2^ g^−1^ owing to the removal of the aromatic CO- and phenolic OH linked to aromatic cores at high pyrolysis temperature. The high S_BET_ of WH300, WH500, and WH700 indicated fine pore structures, which contributed to their adsorptive effects.

**Table 2:** Physicochemical properties of biochars pyrolyzed at different temperature.

Biochar	S_BET_(m^2^/g)	S_micro_(m^2^/g)	S_total_(cm^3^/g)	V_micro_(cm^3^/g)	D_ap_(nm)
**WH300**	3.5150	0.7417	0.0068	0.0005	7.7665
**WH500**	6.7131	3.1843	0.0148	0.0019	8.7949
**WH700**	175.4568	134.6544	0.1183	0.0709	2.6971

**Fig 2:**
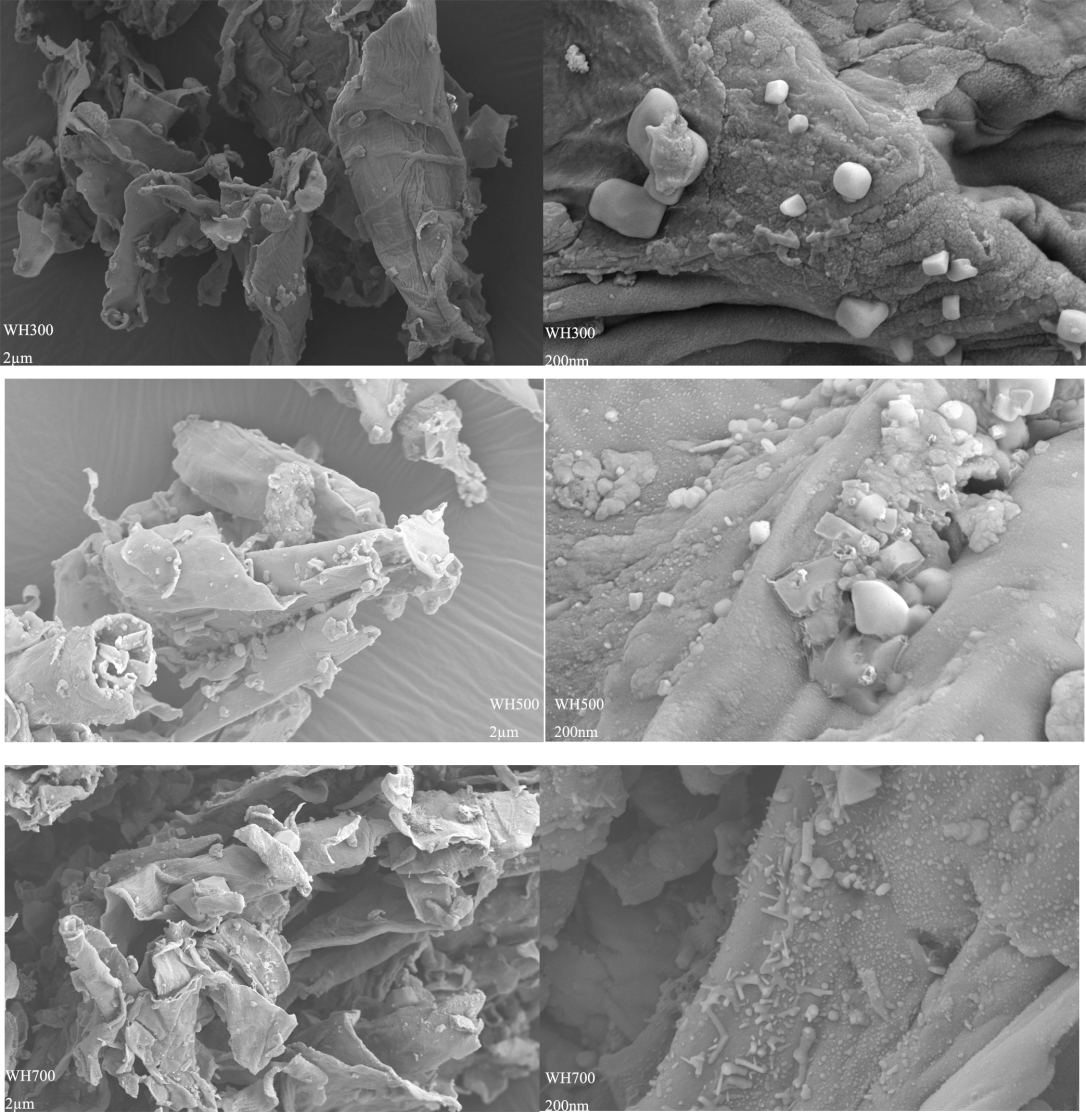
Scanning electron micrographs of biochars pyrolyzed at different temperature.

The SEM images showed that the biochars featured irregular and porous surfaces, although the pore volumes in WH700 were relatively high (Fig 2). These characteristics were consistent with the S_BET_ results.

**Fig 3:**
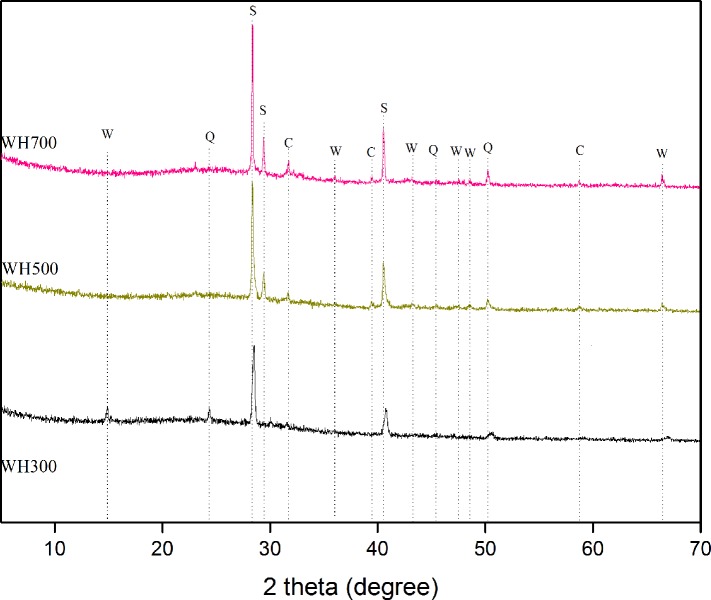
XRD patterns of biochars pyrolyzed at different temperature.

To perform solid component characterization, the biochar samples without Cd treatment were analyzed with XRD. Calcite and whitlockite dominated the surface elements, as shown in the overall XPS spectra (Fig 3). The presence of calcite was consistent with the alkalinity of the biochars (Table 1).

### 3.2 Effect of initial solution pH

**Fig 4:**
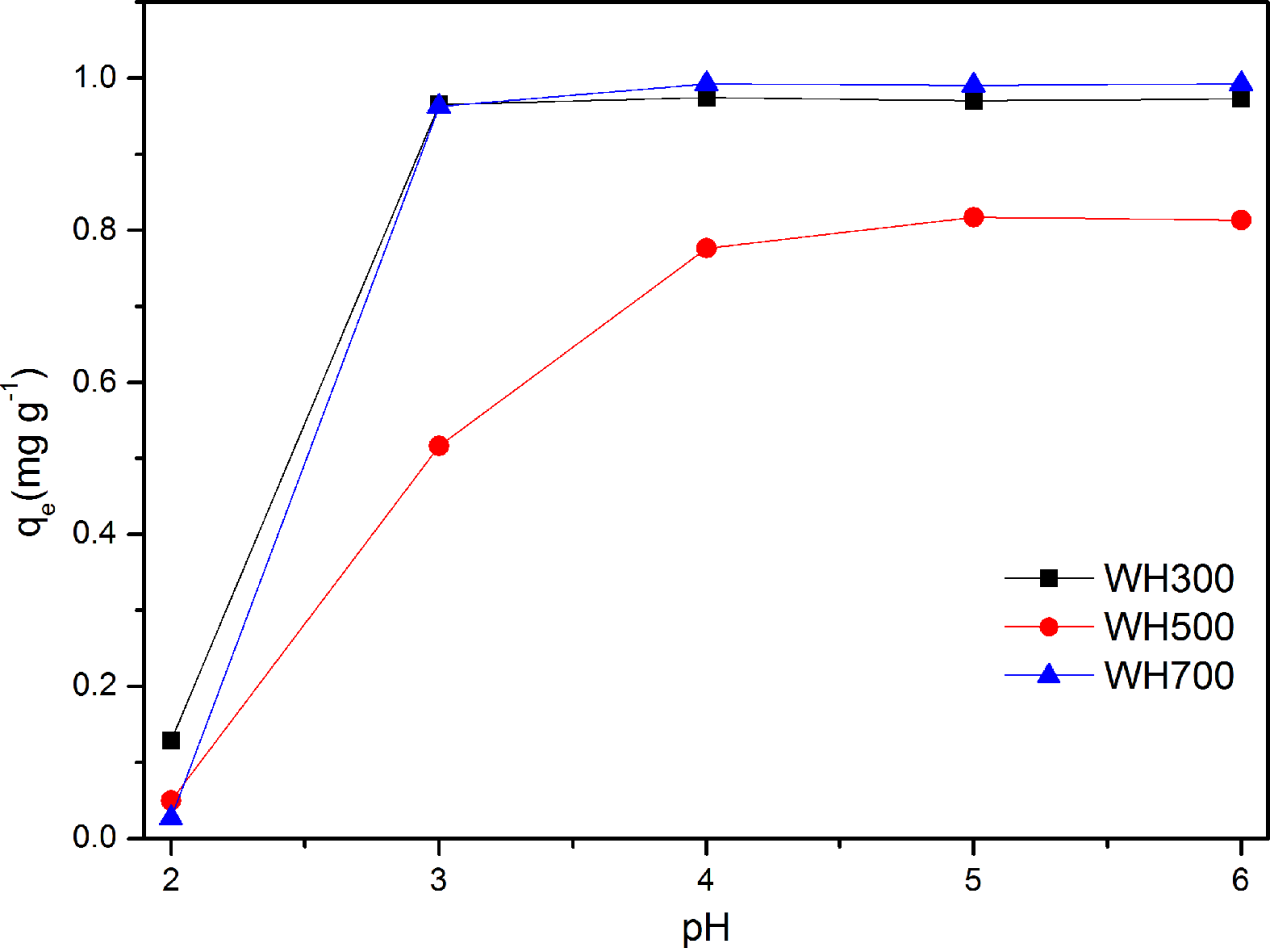
Effect of initial pH on Cd adsorption on WH300, WH500, and WH700.

The effect of the initial solution pH on the Cd adsorption capacities of WH300, WH500, and WH700 is shown in Fig 4. Solution pH is considered as the most important factor in the adsorption of metal onto adsorbents. The effects of initial pH on adsorption capacity are as follows: electrostatic repulsion between the adsorbent and the adsorbate and ion exchange process between the adsorbent and the adsorbate[18]. The Cd adsorption capacities of the biochars increased as the pH of the solution increased from 3.0 to 6.0. The low pH of 2.0 demonstrated an inhibitory effect on the adsorption capacities, whereas the relatively high pH showed a different effect. Moreover, the effect of pH on adsorption capacity is more significant in WH300 than in WH500 and WH700. When the pH of the solution reached 3.0, the adsorption equilibrium of WH500 and WH700 was reached. However, the inhibitory effect on WH300 was observed until the pH value peaked at 5.0, which suggested that the adsorption of Cd onto the biochars was largely influenced by the pH level of the aqueous solutions. The effect of pH could be explained by considering the surface charge on the adsorbent [9]. At a low pH, the surfaces of the biochars were positively charged. Hence, the Cd adsorption capacity would be quite low because of the high electrostatic repulsion between metallic ions [7,9]. Furthermore, hydrogen ions would strongly compete for adsorption sites with Cd ions, resulting in the inhibitory effects on adsorption capacity[19]. With increased pH value, the deprotonation of the functional groups provided the chance to coordinate with Cd ions and led to high removal rates. At a high pH (5.0 to 6.0), the Cd species would begin to hydrolyze as Cd (OH)^+^, which improves the absorption capacity of biochars[15]. The Cd sorption capacity of WH300 was much higher than that of WH500 at same pH despite its lower specific surface area (Table 1), suggesting the Cd sorption was not only controlled by its surface area. Besides, WH700 has almost same Cd sorption capacity of WH300,which may governed by ash content and exchangeable ions in the adsorption process[20].

In our experiment, the Cd adsorption capacities of WH300, WH500, and WH700 were 1.917, 1.582 and 1.976 mg g^−1^, respectively, at a pH value 5.0. In the following adsorption experiments, a pH of 5.0 was selected as the optimum pH value.

### 3.3 Effect of initial concentration and adsorption isotherms

**Fig 5:**
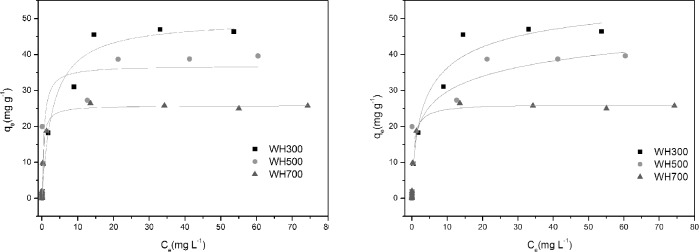
Adsorption isotherms of Cd on WH300, WH500, and WH700.

Fig 5 presents the isotherms of the Cd adsorption of the biochars with different initial Cd concentrations ranging from 0.01 mg L^−1^ to 50 mg L^−1^. The Cd adsorption capacity of the biochars increased rapidly at low initial concentrations and then increased slowly with the Cd concentrations of 10 mg L^−1^ (for WH300), 15 mg L^−1^ (for 500°C), and 20 mg L^−1^ WH700) until the adsorption reached equilibrium.

Table 3 presents the parameters and correlation coefficients for the adsorption obtained by regressing the experimental data. The high R^2^ of the models indicated that the data fitted the models satisfactorily. As can be seen from the isotherms and regression coefficients, both models fitted the experimental data fairly well with correlation coefficients (R^2^) above 0.90 (Table 2). The R^2^ values indicated that the Freundlich isotherm model fitted the experimental data better than the Langmuir isotherm model did (R^2^ ≥ 0.90 for the Freundlich model). Such result suggested that the adsorption of Cd onto the biochars could be attributed to the mechanisms of Freundlich surface adsorption. The Langmuir constants k_L_ of WH300, WH500 and WH700 were 0.328, 1.856, and 2.683 L mg^−1^ respectively. The maximum Cd adsorption capacities obtained with the Langmuir model were 49.837, 36.899, and 25.826 mg g^−1^ for WH300, WH500, and WH700, respectively (Table 3). The removal efficiency (%) of Cd by three biochars was all over 90%, in which maximum removal efficiency reached 99.24% (WH700 concentration 1 g L^−1^; initial Cd concentration 1 molL^-l^; reaction temperature 25°C; initial solution pH 5.0)

**Table 3:** Kinetic parameters for Cd adsorption on WH300, WH500, and WH700.

Biochar	Langmuir model			Freundlich model		
	q_e_(mgg^-1^)	K_L_ (L mg^−1^)	R^2^	K_F_ (mg^1−1/n^l^1/n^ g^−1^)	n	R^2^
**WH300**	49.837	0.328	0.9835	3.871	0.541	0.9837
**WH500**	36.899	1.856	0.8887	5.194	0.301	0.9035
**WH700**	25.826	2.683	0.9972	7.046	0.246	0.9978

Other studies indicated that biochars produced at high temperatures possess higher adsorption capacities of heavy metals compared with those produced at low temperatures [21,22].However, opposite results were observed in the present experiment. Biochars produced at low temperatures tend to have numerous oxygen-containing functional groups, such as carboxyl and hydroxyl. Such characteristic is consistent with the FTIR results in the present study. These oxygen-containing functional groups can serve as effective sorption sites for metal ions via complexation[23,24]. A comparison of the maximum Cd adsorption capacities of biochars derived from E. crassipes with biochars from other biomass are presented in Table 4.

**Table 4:** Comparison of Cd adsorption capacity (Q_max_) of biochars from *E*. *crassipes* with other biomass-based biochars in literature.

Biomass	PyrolysisTemperature(°C)	Absorption condition		Q_max_(mg g^-1^)	Reference
		**pH**	**Temperature(°C)**		
buffalo weed	700	6.0	25	13.369	Roh.et.al 2015[25]
hickory wood	600	NA	22 ± 0.5	28.1	Wang et.al 2015[8]
bamboo	450	5.80	25±2	11.51	Tan et.al 2015[26]
coconut shell	450	5.26	25±2	9.07	Tan et.al 2015[26]
pine woodshavings	450	5.72	25±2	10.37	Tan et.al 2015[26]
sugarcane bagasse	450	5.36	25±2	9.91	Tan et.al 2015[26]

### 3.4 Effect of contact time and adsorption kinetics

**Fig 6:**
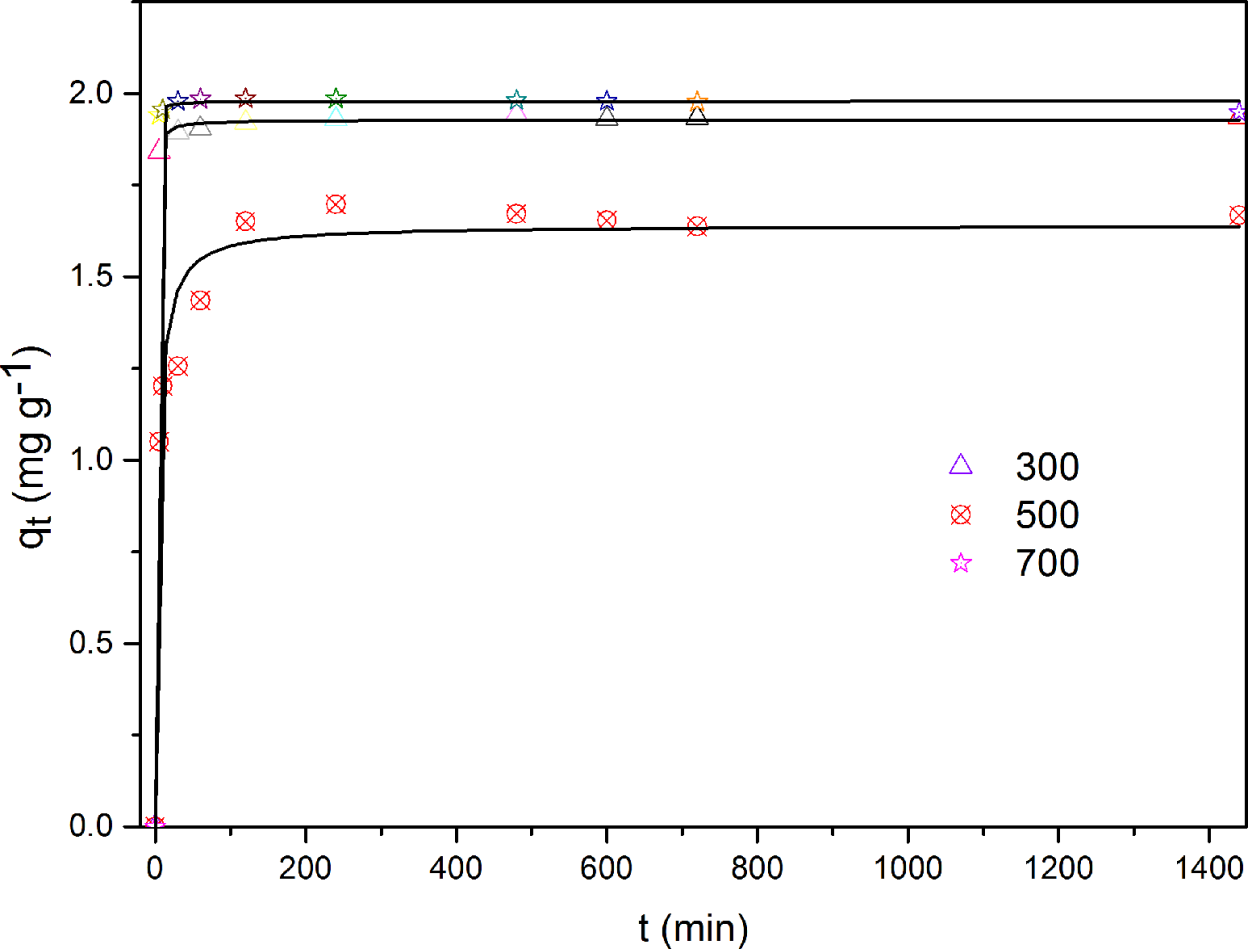
Adsorption kinetics of Cd on WH300, WH500, and WH700.

The effect of contact time on the adsorption capacities of WH300, WH500, and WH700 is presented in Fig 6. The rates of the sorption of Cd were rapid during the initial 30 min, with over 70% of Cd ions removed. The rates then increased slowly as the equilibrium approached 24 h. A contact time of 24 h was used across all the subsequent experiments to ensure that adsorption equilibrium was reached.

The kinetics parameters and correlation coefficients (R^2^) of the three models are presented in Table 5, which shows that compared with the pseudo-first-order model and Elovich model, the pseudo-second-order model showed a good correlation between the experimental results and the R^2^ values of WH300 and WH700 above 0.999. The results indicated that the kinetic behavior of Cd on biochars can be satisfactorily explained with the pseudo-second-order model, in which case the rate-limiting step may be chemical sorption rather than diffusion. The equilibrium adsorption capacities (q_e_) obtained with the pseudo-second-order model was consistent with the experimental value (q_e_) of 1.979 mg/g.

**Table 5:** Isotherm parameters for Cd adsorption on WH300, WH500, and WH700.

Biochar	Q_e_(mg/g)	Pseudo-first-order model			Pseudo-second-order model			Elovich model		
		q_e_(mg/g)	k_1_(h^-1^)	R^2^	q_e_(mg/g)	K_2_(mg/(g·h))	R^2^	α(mg/(g·h))	β(g/mg)	R^2^
**WH300**	1.931	1.917	0.634	0.9979	1.928	1.849	0.9994	1.08E43	56.734	0.9995
**WH500**	1.64	1.582	0.178	0.9165	1.640	0.169	0.9656	359.350	8.608	0.9694
**WH700**	1.979	1.976	0.807	0.9995	1.979	5.242	0.9996	1.26E46	58.342	0.9973

The best fit k_1_ (first-order apparent adsorption rate constants) was 0.807 h^−1^, which indicated that half of the available adsorption sites on the adsorbent could be filled with Cd in 1.2 h. The adsorption capacities of Cd(II) onto WH300, WH500, and WH700 calculated with the pseudo-second-order model were 1.928, 1.640, and 1.979 mg g^−1^, respectively. By using the rate constants of the pseudo-second-order model for comparative purposes, we arrived at the following adsorption rates: WH700 (5.242) > WH300 (1.849) > WH500 (0.169). Comprehensively considering the adsorption capacity at equilibrium, the WH300 can be regarded as a good adsorbent for the effective removal of Cd from aqueous solution.

## Conclusions

According to the results of the elemental and Fourier transform infrared spectroscopy analyses, the adsorption of Cd onto the biochars was dominated by oxygen-containing functional groups via esterification reactions. The X-ray diffraction results indicated that the mineral components contributed to absorbing Cd through precipitation or co-precipitation. The maximum adsorption capacities based on the Langmuir isotherm were calculated to be 49.837, 36.899, and 25.826 mg g^−1^. The adsorption processes followed the pseudo-second-order kinetics, with the equilibrium achieved around 5 h. The biochar from *E*. *crassipes* is a promising adsorbent for the treatment of wastewater, which can in turn convert one environmental problem to a new cleaning Technology.
